# Impact of Post-Procedural Change in Left Ventricle Systolic Function on Survival after Percutaneous Edge-to-Edge Mitral Valve Repair

**DOI:** 10.3390/jcm10204748

**Published:** 2021-10-16

**Authors:** Magnus J. Hagnäs, Carmelo Grasso, Maria Elena Di Salvo, Anna Caggegi, Marco Barbanti, Salvatore Scandura, Annalisa Milici, Gessica Motta, Agnese Bentivegna, Andrea Sardone, Luigi Capodicasa, Angelo Giuffrida, Fausto Biancari, Timo Mäkikallio, Davide Capodanno, Corrado Tamburino

**Affiliations:** 1 Department of Internal Medicine, Heart Smithy, Lapland Central Hospital, 96101 Rovaniemi, Finland; 2 Division of Cardiology, CAST, P.O. “Rodolico”, Azienda Ospedaliero-Universitaria, “Policlinico-Vittorio Emanuele”, University of Catania, 95123 Catania, Italy; melfat75@gmail.com (C.G.); marilena77@tiscali.it (M.E.D.S.); caggegi_ouy@yahoo.it (A.C.); mbarbanti83@gmail.com (M.B.); salvatore.scandura@tin.it (S.S.); Miliciannalisa@gmail.com (A.M.); mottagessica@gmail.com (G.M.); bentivegnaagnese@gmail.com (A.B.); andreasar91@gmail.com (A.S.); capodicasaluigi@gmail.com (L.C.); angelo.giuffrida@unict.it (A.G.); dcapodanno@gmail.com (D.C.); tambucor@unict.it (C.T.); 3 Cardiac Surgery, Clinica Montevergine, GVM Care & Research, 83013 Mercogliano, Italy; faustobiancari@yahoo.it; 4 Cardiac Surgery, Helsinki University Hospital, 00280 Helsinki, Finland; 5 Department of Medicine, University of Helsinki, 00100 Helsinki, Finland; timo.makikallio@ppshp.fi; 6 South-Karelia Central Hospital, 53130 Lappeenranta, Finland

**Keywords:** MitraClip, heart failure, left ventricle ejection fraction, secondary mitral regurgitation, mortality

## Abstract

Objectives: To investigate how the changes of left ventricle ejection fraction (LVEF) between admission and discharge affected the long-term outcome in patients who underwent percutaneous edge-to-edge mitral valve repair for secondary mitral regurgitation. Background: An acute impairment of LVEF after surgical repair of mitral regurgitation, known as afterload mismatch, has been associated with increased all-cause mortality. Afterload mismatch after percutaneous edge-to-edge mitral valve repair has been postulated to be a transient phenomenon. Methods: This study is based on a single-center, retrospective, observational registry of patients who underwent percutaneous edge-to-edge mitral valve repair with the MitraClip (Abbot Vascular) system for the treatment of symptomatic, moderate-to-severe mitral regurgitation. We included data on 399 patients who underwent percutaneous edge-to-edge mitral valve repair for secondary mitral regurgitation. Expert echocardiographers assessed LVEF before the procedure and at discharge. The patients were divided into three groups according to the difference of periprocedural LVEF measurements: unchanged (*n* = 318), improved (*n* = 40), and decreased (*n* = 41) LVEF. Results: The median follow-up time was 2.0 years. When adjusted for gender, NYHA class and estimated glomerular filtration rate, decreased postprocedural LVEF was associated with an increased risk of death (adjusted HR 2.05, 95% CI 1.26–3.34) and increased postprocedural LVEF with a reduced risk of death (adjusted HR 0.47, 95% CI 0.24–0.91) compared to unchanged LVEF. **Conclusion:** Among patients who underwent percutaneous edge-to-edge mitral valve repair, decreased postprocedural LVEF was associated with increased mortality, while improved LVEF was associated with lower mortality compared to unchanged LVEF.

## 1. Introduction

Acute impairment of left ventricle ejection fraction (LVEF) after surgical repair of mitral regurgitation (MR), known as afterload mismatch, has been shown to increase postoperative mortality [1]. Previously, afterload mismatch occurring after transcatheter mitral valve repair has been postulated to be a transient phenomenon [2], but recent studies demonstrated its negative prognostic effect [3]. Still, the impact of periprocedural changes of LVEF after transcatheter mitral valve repair is mostly unknown.

Moderate-to-severe MR causes progressive left ventricle dysfunction and congestive heart failure [4]. Prolonged alternation in loading conditions caused by MR can lead to adverse left ventricle remodeling, which over time, carries a poor prognosis [5]. The initial treatment strategy for MR and heart failure is drug treatment [6]. However, patients with symptoms despite guideline-directed medical therapy may require transcatheter or surgical mitral valve repair or replacement. In most patients, significant improvements in LVEF can be achieved by mitral valve repair [7]. Still, the more advanced the heart failure is, the worse is the long-term survival after mitral valve repair [8], because of irreversible adverse modeling of the left ventricle and, in some cases, of acute afterload mismatch. By identifying the patients at a higher risk of afterload mismatch, we could implement potential pharmacologic support during the procedure [9,10].

In this study, we sought to investigate the prognostic impact of post-procedural changes of LVEF on the long-term outcome among patients who underwent percutaneous edge-to-edge mitral valve repair with the MitraClip (Abbot Vascular, Santa Clara, California) system for secondary MR.

## 2. Materials and Methods

This study is based on the Getting Reduction of mitrAl inSufficiency by Percutaneous clip implantation (GRASP) registry, which is a retrospective, observational registry on patients who underwent MitraClip implantation for the treatment of symptomatic, moderate-to-severe MR at the Division of Cardiology, A.O.U. “Policlinico-Vittorio Emanuele”, University of Catania, Catania, Italy. The study population comprised 399 consecutive patients who underwent percutaneous edge-to-edge mitral valve repair with the MitraClip system for secondary MR between October 2008 and December 2019. This study conforms to the principles of the Declaration of Helsinki. Baseline, operative, and outcome data were entered into a dedicated database and were assessed for quality and completeness. A multidisciplinary heart team, including a cardiac surgeon, an interventional cardiologist, a clinical cardiologist, and an anesthesiologist, decided regarding the indication for percutaneous edge-to-edge mitral valve repair following current guidelines. No specific exclusion criteria were adopted. Two expert echocardiographers quantified LVEF, using the biplane Simpson method, left ventricle end-diastolic diameter (LVEDD), and the degree of pre-procedural MR based on current recommendations [11]. Technical and procedural details of the percutaneous edge-to-edge mitral valve repair have been previously reported [12]. All procedures were performed in a standard catheterization laboratory, with surgical backup, with transesophageal guidance and under general anesthesia (except one patient was treated under local anesthesia). The estimated glomerular filtration rate (eGFR) was calculated using the Modification of Diet in Renal Disease Study equation: GFR (mL/min/1.73 m^2^) = 175 × (Creatinine in mg/Dl)^−1.154^ × (Age)^−0.203^ × (0.742 if female). Guideline-directed medical therapy was defined as the use of appropriate classes of neurohormonal antagonists for heart failure or not: either angiotensin-converting enzyme inhibitors or angiotensin receptor blockers, beta-blockers, and, in patients with a left ventricular ejection fraction of 35% or less, mineralocorticoid receptor antagonist at discharge. All patients gave their informed consent to the procedure, data collection, and reporting in an aggregated and anonymized form. This study was approved by the ethics committee at the Ferrarotto Hospital, University of Catania, Italy.

All outcomes of interest for this study were defined according to Mitral Valve Academic Research Consortium criteria [13]. The primary outcome of interest was all-cause mortality. The secondary outcome of interest was death from cardiovascular causes, including sudden death or death due to heart failure, myocardial infarction, stroke, arrhythmia, or periprocedural causes. Scheduled clinical evaluations and phone interviews prospectively collected follow-up data. Referring cardiologists, general practitioners, and patients were contacted whenever additional information was needed.

Patients were stratified by the post-procedural change of at least 1% in LVEF into three groups according to the difference of LVEF measurements before and after the procedure (either the next day or before hospital discharge): unchanged (*n* = 318), improved (*n* = 40), and decreased (*n* = 41) LVEF. Continuous variables that are normally distributed are presented as means and standard deviation (SD), while those not normally distributed are presented as the median and interquartile range (IQR). Continuous variables were compared using Kruskal–Wallis test or the Mann–Whitney *U* test. Dichotomous parameters are presented as counts and percentages and compared using the chi-square test. Cumulative event rates were estimated with the Kaplan–Meier method, and a comparison of clinical outcomes was performed using the log-rank test. A logistic regression was performed to analyze predictors of post-procedural decrease in LVEF. A multivariable Cox proportional hazards survival model was used to analyze the risk of death. Covariates to be entered into the multivariable model were selected according to statistical significance in univariate analysis. These included gender, the New York Heart Association (NYHA) class, and eGFR. For all analyses, a 2-sided *p* value <0.05 was considered significant. Statistical analyses were performed using Statistical Package for Social Sciences version 25.0 (IBM Corporation, Armonk, NY, USA) and Software for Statistics and Data Science version 15.1 (StataCorp LLC, College Station, TX, USA) statistical software.

## 3. Results

**Figure 1:**
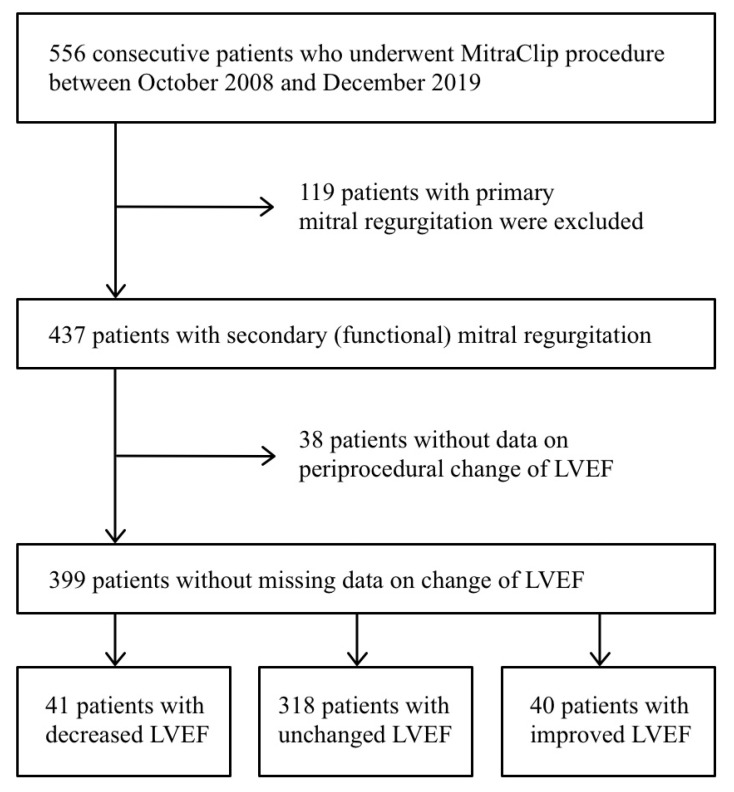
Study flow-chart.

Data on post-procedural change in LVEF were available for 399 of the 437 (91%) patients who underwent the percutaneous edge-to-edge mitral valve repair for secondary MR (Figure 1). Data on survival were available at 1-, 2- and 3-year follow-up in 338 (93.6% of 361), 288 (90.6% of 318), and 213 (78.6% of 271) of the patients, respectively. Table 1 presents the baseline characteristics of the study population. The mean age was 72.6 years, and 60% (N = 239) of the patients were males. A comparison of baseline characteristics according to post-procedural change in LVEF revealed that patients with improved LVEF had a lower mean LVEF (26% vs. 35%, *p* < 0.001), as well as not statistically significant higher NT-Pro B-type natriuretic peptide levels (median, 1087 vs. 787 ng/mL, *p* = 0.553) and higher EuroSCORE II (median, 7.2% vs. 6.1%, *p* = 0.541) compared to patients with unchanged LVEF. Before the procedure 185 (46%) patients had atrial fibrillation, which was not a significant risk factor for all cause-mortality (*p* = 0.382). The overall adherence to guideline-directed medical therapy was 38% (137 of 361), and the proportions were similar among the patients with a different post-procedural change in LVEF (*p* = 0.854), Supplementary Table S1. Ischemic etiology was diagnosed for 206 (52%) of the patients; there was no statistically significant difference in the outcome compared to the patients without ischemic etiology of MR (hazard ratio 0.94 [HR] in univariate analysis, 95% confidence interval [CI] 0.68–1.29, *p* = 0.694 and HR 1.44 in multivariate analysis, 95% CI 0.98–2.06, *p* = 0.054).

**Figure 2:**
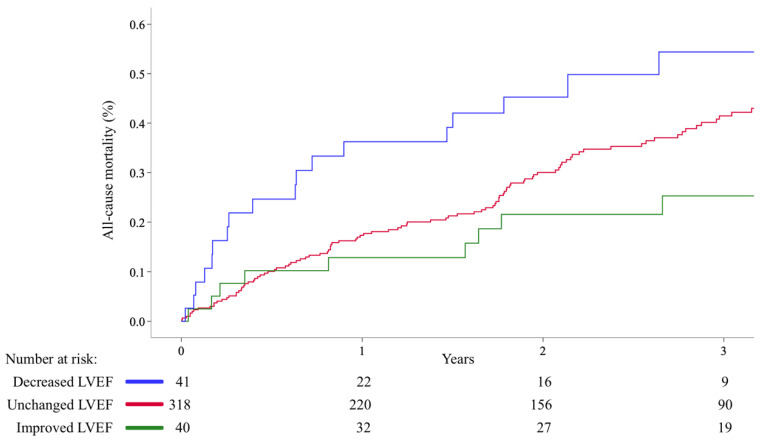
A Kaplan–Meier survival estimate after percutaneous edge-to-edge mitral valve repair among patients divided by post-procedural change in left ventricle ejection fraction (LVEF) (log-rank *p* = 0.011).

During a median follow-up time of 2.0 years, we observed 150 (38%) deaths. Figure 2 shows the incidence of all-cause deaths among patients divided according to post-procedural change in LVEF. The Kaplan–Meier estimates of all-cause mortality in patients with unchanged LVEF at 30-day and 3-year follow-up were 2.3% and 41%; in patients with decreased LVEF, 7.9% and 54%; and in patients with improved LVEF, 2.5% and 25%, respectively. The overall 3-year Kaplan–Meier estimates among all included patients and the patients who were excluded because of missing LVEF data (*n* = 38) were 59.2% and 53.7%, respectively.

**Figure 3:**
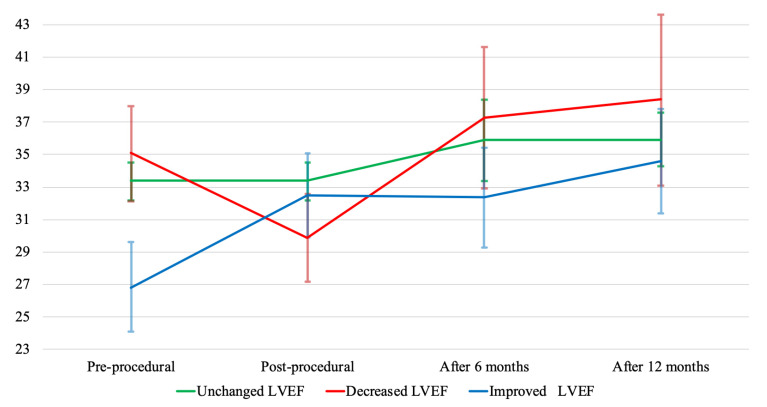
Change in mean left ventricular ejection fraction (LVEF) among patients divided according to post-procedural change in LVEF, with 95% confidence intervals.

When adjusted for other independent risk factors such as gender, NYHA classes, and eGFR, decreased LVEF was associated with a significant increase in the risk of death (adjusted HR 2.05, 95% CI 1.26–3.34, *p* = 0.004), and increased LVEF was associated with a significant decrease in risk of death (adjusted HR 0.47, 95% CI 0.24–0.91, *p* = 0.024) compared to unchanged LVEF. The HRs of univariate and multivariate analysis are shown in Table 2. Figure 3 shows the follow-up changes in LVEF among patients stratified according to post-procedural change in LVEF. LVEF and LVEDD improved among all patients during the 6- and 12-month follow-ups, particularly among those with post-procedural decreased LVEF (Table 3). Age, atrial fibrillation, and LVEF were statistically significant predictors of decreased post-procedural LVEF (*p* = 0.012, 0.009 and 0.019, respectively).

Among 264 patients with low pre-procedural LVEF (35% or less), 106 (39%) deaths occurred. A decreased LVEF was associated with an increased risk of all-cause mortality (adjusted HR 3.10, 95% CI 1.72–5.61, *p* < 0.001), while improved LVEF had a similar mortality risk (adjusted HR 0.70, 95% CI 0.35–1.41, *p* = 0.314) compared to unchanged LVEF. Among the 135 patients with pre-procedural LVEF of more than 35%, 25 (18%) deaths occurred. Among these patients, the post-procedural change in LVEF did not affect the risk of mortality. The cumulative incidence of all-cause mortality among patients with pre-procedural LVEF ≤ 35% and LVEF > 35%, divided according to post-procedural change in LVEF, is shown in Supplemental Figure S1.

A total of 102 (30%) deaths from cardiovascular causes were observed. A decreased LVEF was associated with an increased risk of cardiovascular mortality (adjusted HR 2.05, 95% CI 1.15–3.65, *p* = 0.014), while improved LVEF had a similar mortality risk (adjusted HR 0.62, 95% CI 0.29–1.29, *p* = 0.199) compared to unchanged LVEF.

## 4. Discussion

The main findings of this study were that a post-procedural improvement in LVEF was associated with a decreased risk of all-cause mortality, and a decrease in post-procedural LVEF was associated with an increased risk of all-cause mortality. The mean LVEF improved, and LVEDD decreased during follow-up in all patient groups. These findings indicate that patients with MR and reversible heart failure benefit from percutaneous edge-to-edge mitral valve repair.

Correcting the MR redirects the low impedance regurgitant flow into the aorta, where the pressure is higher than in the left atrium; this increases the left ventricular afterload. The acute change in loading conditions might cause an impairment of systolic function called afterload mismatch [4,9,14]. A possible explanation for the increase in LVEF after a repair of MR could be that the left ventricle has the potential of positive remodeling, i.e., heart failure is reversible. Instead, a decrease in LVEF could be an indication of irreversible adverse remodeling of the left ventricle, which increases the risk of mortality. Another observation that supports this finding is that, even if the pre-procedural LVEF and follow-up LVEF were the lowest among the patients whose post-procedural LVEF improved, they still had the lowest mortality rate. However, those patients with a decrease in post-procedural LVEF who survived, improved their LVEF the most during follow-up, indicating a reversible heart failure. This highlights the difficulty of identifying patients with reversible heart failure. We observed a positive remodeling of the left ventricle, measured as a reduction in mean LVEDD, in all patient groups, which could indicate that among those who survive, a positive remodeling of the left ventricle occurs.

Previous studies demonstrated that a decrease in LVEF immediately after surgery for MR increases mortality risk [1]. Afterload mismatch has been reported to be observed in 23% to 26% of transcatheter mitral valve repair procedures as well [2,3]. However, studies showed inconsistent findings regarding how the afterload mismatch affects the outcome after transcatheter mitral valve repair. Previous studies reported that afterload mismatch is a transient finding without subsequent long-term effects [2], while in more recent studies, it is associated with poor clinical outcome [3]. Afterload mismatch seems more prominent during a surgical procedure compared to a transcatheter procedure, which can be explained with myocardial ischemia and the use of cardiopulmonary bypass [1,14]. To our knowledge, no previous studies have shown that a decrease in LVEF at discharge impacts the long-term survival of patients who underwent percutaneous edge-to-edge mitral valve repair for secondary MR. Other studies reported worse long-term survival after mitral valve repair in advanced heart failure [8,15,16]. In our study, LVEDD was higher, and LVEF was lower in patients with improved post-procedural LVEF, who subsequently also had the best survival. This finding suggests that patients who experience an increase in LVEF after the percutaneous edge-to-edge mitral valve repair, even if baseline LVEF is low, may still benefit from this procedure. In concert with previous studies, our study demonstrated that in most patients, LVEF improves during follow-up after mitral valve repair [7,17]. An increase in LVEF during echocardiographic stress test might be useful in identifying patients who may benefit most from transcatheter mitral valve repair; however, we need more studies to confirm this finding [18,19].

Identifying patients who may benefit the most from transcatheter mitral valve repair is an issue [20]. According to previous studies, patients with advanced heart failure and very low LVEF might not benefit from transcatheter mitral valve repair [16,21]. However, we should identify among the patients with low LVEF those who have a potential of positive remodeling, which can be seen as post-procedural LVEF improvement. Unfortunately, our study does not provide an answer to that question. We also observed that the risk of death was higher among the patients whose LVEF decreased after percutaneous edge-to-edge mitral valve repair; these patients should be followed up closely, heart failure medication should be optimized, and the need for implantable cardioverter-defibrillators and cardiac resynchronization therapy should be assessed. The patients at a higher risk of afterload mismatch should be identified before the procedure, and potential pharmacological support during the procedure should be considered, such as inotropic support and levosimendan infusion [9,10]. In selected cases, mechanical circulatory support such as a percutaneous left ventricular assist device like the Impella CP (Abiomed, Danvers, MA), or peripheral venoarterial extracorporeal membrane oxygenation [22] could also be considered.

The results of this study have several limitations. Firstly, this was an observational registry, and selection bias could be at play. However, our findings are from a real-world setting and are clinically plausible. Secondly, the study period spanned over 10 years, and management of heart failure might have changed over time. However, adjustment for this did not affect our results. Finally, data were site-reported and not assessed by a core-lab. It could be argued that a slight change of 1% in LVEF cannot be reproduced; however, the present findings show that in a clinical setting, even a slight change in LVEF measured by the echographer may affect the long-term outcome after a percutaneous edge-to-edge mitral valve repair.

## 5. Conclusions

Among patients who underwent a percutaneous edge-to-edge mitral valve repair, decreased post-procedural LVEF was associated with increased mortality, while improved LVEF was associated with lower mortality compared to unchanged post-procedural LVEF. LVEF was lowest among the patients with improved post-procedural LVEF, which demonstrates that not all patients with low pre-procedural LVEF have irreversible adverse remodeling of the left ventricle. This study shows that even the patients with a very low LVEF might benefit from a percutaneous edge-to-edge mitral valve repair for MR if the left ventricle is capable of reverse remodeling.

## Figures and Tables

**Table 1:** Baseline characteristics of patients stratified according to post-procedural change in left ventricular ejection fraction.

	Overall Series	Unchanged LVEF	Decreased LVEF	Improved LVEF	*p*-Value
*n*	399	318	41	40	
Age (years)	72.6 ± 8.9	72.7 ± 8.4	72.9 ± 11.8	71.9 ± 8.9	0.389
Gender (male), n (%)	239 (60)	190 (60)	23 (56)	26 (65)	0.711
Diabetes, n (%)	154 (39)	133 (42)	11 (27)	10 (25)	0.148
Hypertension, n (%)	317 (79)	256 (81)	30 (73)	31 (78)	0.522
Previous CABG, n (%)	84 (21)	64 (20)	8 (20)	12 (30)	0.341
Atrial fibrillation, n (%)	185 (46)	141 (44)	29 (71)	15 (38)	0.003
eGFR (mL/min/1.73 m^2^)	50.6 (35.6–66.8)	50.1 (35.3–66.4)	61.1 (36.4–71.1)	53.5 (42.0–71.1)	0.549
ProBNP (pg/mL)	709 (323–2324)	674 (322–2446)	787 (341–1894)	1087 (320–2044)	0.800
EuroSCORE II	6.2 (3.6–10.8)	6.1 (3.6–10.8)	6.1 (3.8–11.2)	7.2 (3.3–11.3)	0.759
Ischemic MR, n (%)	206 (52)	161 (51)	26 (63)	19 (48)	0.178
ICD-pacemaker, n (%)	100 (25)	78 (25)	41 (22)	40 (33)	0.487
CRT-pacemaker, n (%)	40 (10)	31 (10)	6 (15)	3 (8)	0.529
NYHA II, n (%)	55 (14)	42 (13)	5 (12)	8 (20)	0.478
NYHA III, n (%)	296 (74)	241 (76)	29 (71)	26 (65)	0.295
NYHA IV, n (%)	48 (12)	35 (11)	7 (17)	6 (15)	0.442
LVEF (%)	32.9 ± 10.6	33.4 ± 10.8	35.1 ± 9.3	26.8 ± 8.6	<0.001
MR grade III, n (%)	117 (31)	94 (32)	9 (23)	14 (36)	0.435
MR grade IV, n (%)	255 (69)	200 (68)	30 (77)	25 (64)	0.435
LVEDD (mm)	60.6 ± 10.8	60.2 ± 11.0	61.2 ± 9.6	61.9 ± 9.7	0.378
LVESD (mm)	47.1 ± 12.0	48.8 ± 12.2	47.7 ± 11.4	48.7 ± 11.5	0.710
TAPSE (mm)	18.0 ± 4.2	18.2 ± 4.2	17.2 ± 4.9	17.3 ± 3.4	0.190
PASP (mmHg)	47.0 ± 13.2	46.7 ± 12.9	47.9 ± 13.3	48.3 ± 16.2	0.716
Left atrial area (cm^2^)	27.8 ± 8.4	27.4 ± 8.1	30.1 ± 10.2	28.3 ± 9.1	0.343

Normally distributed data are presented as means and ± standard deviation (SD). Abnormally distributed data are presented as median and interquartile range (IQR). LVEF = left ventricle ejection fraction; CABG = coronary artery bypass grafting; eGFR = pre-procedural estimation of glomerular filtration rate calculated using the Modification of Diet in Renal Disease Study equation; proBNP = NT-Pro B-type natriuretic peptide; MR = mitral regurgitation; ICD = implantable cardioverter defibrillator; CRT = cardiac resynchronization therapy; NYHA = New York Heart Association; LVEDD = left ventricle end diastolic dimension; LVESD = left ventricle end systolic diameter; TAPSE = tricuspid annular plane systolic excursion; PAPs = pulmonary arterial systolic pressure.

**Table 2:** Predictors of mortality.

Variable	Univariate Analysis		Multivariable Analysis	
	HR (95% CI)	*p* Value	HR (95% CI)	*p* Value
Gender (male)	1.43 (1.02–2.01)	0.038	1.62 (1.15–2.29)	0.006
NYHA Class III	1.52 (0.87–2.65)	0.144	1.26 (0.72–2.22)	0.419
NYHA Class IV	4.18 (2.22–7.87)	<0.001	3.88 (2.05–7.37)	<0.001
eGFR	0.98 (0.98–0.99)	<0.001	0.98 (0.98–0.99)	<0.001
LVEF decreased	1.59 (0.98–2.56)	0.062	2.05 (1.26–3.34)	0.004
LVEF improved	0.53 (0.29–0.99)	0.045	0.47 (0.24–0.91)	0.024

HR = hazard ratio; CI = confidence interval; LVEF = left ventricle ejection fraction; NYHA = New York Heart Association.

**Table 3:** Changes in echocardiographic parameters among patients divided according to post-procedural change in left ventricular ejection fraction.

	Unchanged LVEF	Decreased LVEF	Improved LVEF	*p*-Value	*n* (%)
Pre-procedural LVEF, %	33.4 ± 10.8	35.1 ± 9.3	26.8 ± 8.6	<0.001	399 (100)
Post-procedural LVEF, %	33.4 ± 10.8	29.9 ± 8.6	32.5 ± 8.1	0.251	399 (100)
LVEF after 6 months, %	34.9 ± 10.4	37.3 ± 9.0	32.4 ± 7.8	0.287	235 (59)
LVEF after 12 months, %	35.9 ± 10.6	38.4 ± 10.2	34.6 ± 7.4	0.558	199 (50)
Pre-procedural LVEDD, mm	60.2 ± 11.0	61.2 ± 9.6	62.9 ± 9.7	0.378	357 (89)
LVEDD at 6 months, mm	58.2 ± 9.4	56.1 ± 7.8	62.5 ± 10.2	0.084	229 (57)
LVEDD at 12 months, mm	57.6 ± 10.2	53.5 ± 6.2	61.9 ± 7.7	0.012	189 (47)

Values are mean ± standard deviation. LVEF = left ventricle ejection fraction, LVEDD = left ventricle end-diastolic dimension.

## Data Availability

Not available.

## Supplementary Materials

The following are available online at https://www.mdpi.com/article/10.3390/jcm10204748/s1, Figure S1: A Kaplan–Meier survival estimate after percutaneous edge-to-edge mitral valve repair among patients with (A): low pre-procedural left ventricle ejection fraction (LVEF) ≤ 35% and (B): pre-procedural LVEF > 35% divided by post-procedural changes in LVEF. Table S1: Proportions of patients on guideline-directed medical therapy.

## Abbreviations and Acronyms

CI	confidence interval
eGFR	estimated glomerular filtration rate
GRASP	the Getting Reduction of mitrAl inSufficiency by Percutaneous clip implantation registry
HR	hazard ratio
IQR	interquartile range
LVEDD	left ventricle end-diastolic diameter
LVEF	left ventricle ejection fraction
MR	mitral regurgitation
NYHA	New York Heart Association
SD	standard deviation
