# Supplementary material for: Impact of Post-Procedural Change in Left Ventricle Systolic Function on Survival after Percutaneous Edge-to-Edge Mitral Valve Repair
Source: J Clin Med. 2021 Oct 16;10(20):4748. doi: 10.3390/jcm10204748 (PMC8537749; doi:10.3390/jcm10204748)
Supplement: Supplementary file 1 [file jcm-10-04748-s001.zip › jcm-1391046-supplementary.pdf]

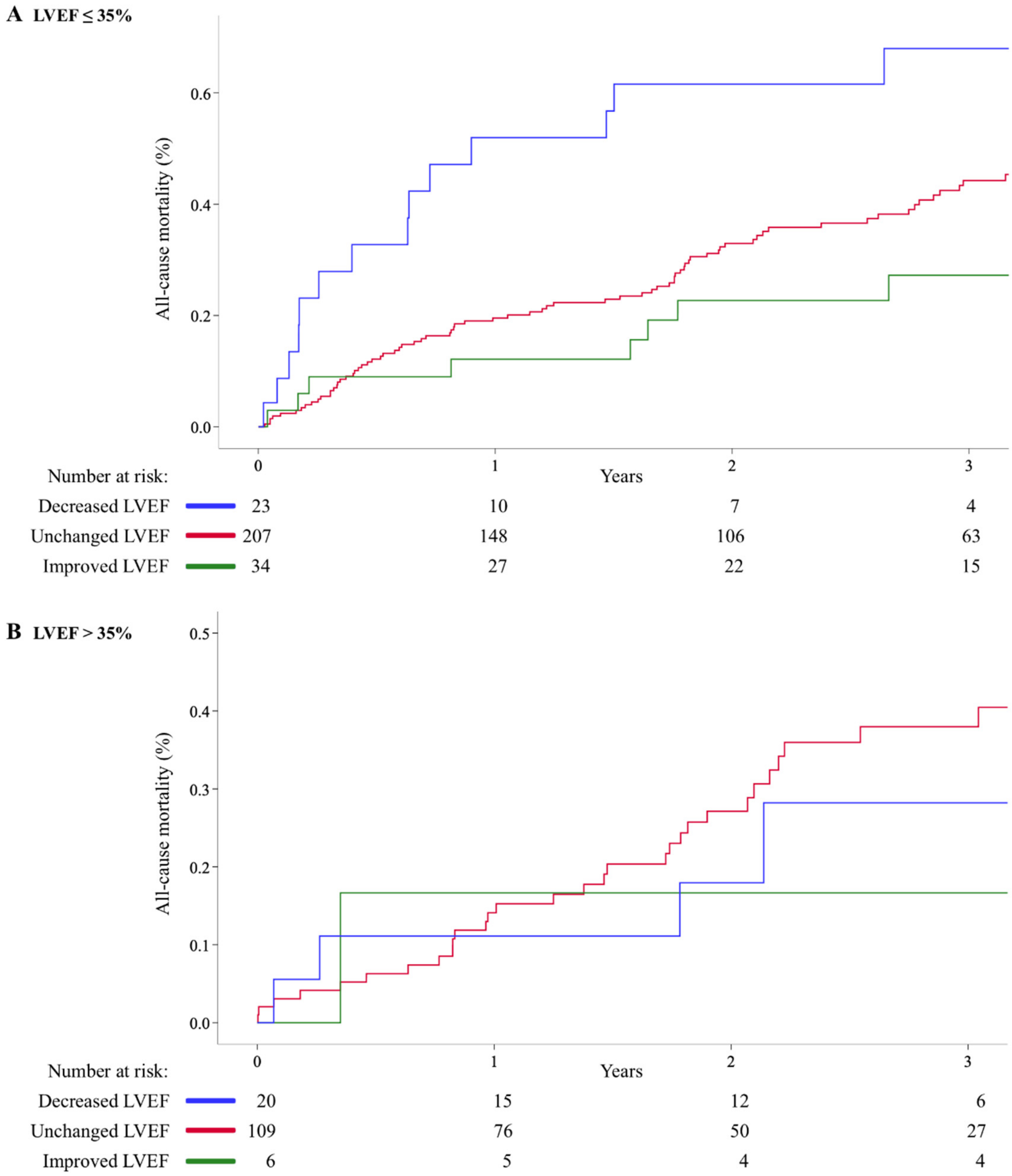

Supplementary Figure S1. A Kaplan–Meier survival estimate after percutaneous edge-to-edge mitral valve repair among patients with (**A**): low pre-procedural left ventricle ejection fraction (LVEF) ≤ 35% and (**B**): pre-procedural LVEF > 35% divided by post-procedural changes in LVEF (log-rank A: $p = 0.003$ B: $p = 0.242$).
